# Embrace, a model for integrated elderly care: study protocol of a randomized controlled trial on the effectiveness regarding patient outcomes, service use, costs, and quality of care

**DOI:** 10.1186/1471-2318-13-62

**Published:** 2013-06-19

**Authors:** Sophie LW Spoorenberg, Ronald J Uittenbroek, Berrie Middel, Berry PH Kremer, Sijmen A Reijneveld, Klaske Wynia

**Affiliations:** 1 Department of Health Sciences, Community and Occupational Medicine, University Medical Center Groningen, University of Groningen, P.O. BOX 196, 9700 AD, Groningen, The Netherlands; 2 Department of Neurology, University Medical Center Groningen, University of Groningen, Groningen, P.O. BOX 30.001, 9700 RB, Groningen, The Netherlands

**Keywords:** Integrated health care systems, Population-based care, Chronic Care Model, Ageing, Chronic disease, Patient outcomes, Quality of care, Cost effectiveness

## Abstract

**Background:**

Ongoing growth in health care expenditures and changing patterns in the demand for health care challenge societies worldwide. The Chronic Care Model (CCM), combined with classification for care needs based on Kaiser Permanente (KP) Triangle, may offer a suitable framework for change. The aim of the present study is to investigate the effectiveness of Embrace, a population-based model for integrated elderly care, regarding patient outcomes, service use, costs, and quality of care.

**Methods/Design:**

The CCM and the KP Triangle were translated to the Dutch setting and adapted to the full elderly population living in the community. A randomized controlled trial with balanced allocation was designed to test the effectiveness of Embrace. Eligible elderly persons are 75 years and older and enrolled with one of the participating general practitioner practices. Based on scores on the INTERMED-Elderly Self-Assessment and Groningen Frailty Indicator, participants will be stratified into one of three strata: (A) robust; (B) frail; and (C) complex care needs. Next, participants will be randomized per stratum to Embrace or care as usual. Embrace encompasses an Elderly Care Team per general practitioner practice, an Electronic Elderly Record System, decision support instruments, and a self-management support and prevention program – combined with care and support intensity levels increasing from stratum A to stratum C. Primary outcome variables are patient outcomes, service use, costs, and quality of care. Data will be collected at baseline, twelve months after starting date, and during the intervention period.

**Discussion:**

This study could provide evidence for the effectiveness of Embrace.

**Trial registration:**

The Netherlands National Trial Register NTR3039

## Background

Societies worldwide are challenged by the ongoing growth in health care expenditures and the changing patterns in the demand for health care. Long-term care expenditures continue to grow and are expected to double within the coming decades [1,2] and the number of elderly people with multiple chronic conditions is increasing drastically [3,4]. More than 50% of people aged 60 years and older suffer from multiple chronic conditions [5] and this percentage will further increase in the coming years [4,6].

Contemporary health care systems face difficulties in solving these challenges, as they have originally been designed to solve single, acute, and mainly short-term diseases [7]. Associated ongoing specialization and technological improvements have led to fragmentation of care delivery and resulted in a substantial increase in health care expenditures. In addition, structural and financial barriers have further increased the segmentation of organizations that provide primary and secondary care, health care, and social care [1,3,8]. Moreover, this fragmentation of care negatively affects the provision of integrated long-term care and support for the chronically ill and for elderly people with complex care needs [5,9]. Despite the wide array of health services they use, these patients do not always receive appropriate and coherent care [4,10,11]. This often leads to adverse drug events, difficulties with participation in treatment, and even treatment errors [4,8,12]. Consequently, health care systems need to be transformed [3,7,8].

Integrated care models promise to provide a solution to control these health care challenges [13,14]. For designing such integrated care models, the Chronic Care Model (CCM) [15] provides a solid and evidence based framework, as acknowledged by the World Health Organization [16]. The CCM was developed to transform the health care system into a system that is equipped for chronic diseases by offering proactive, patient-centered, and integrated care. The model combines community organizations with the health care system and has four evidence-based and interdependent key elements. The four key elements are:

I *Self-management support:* helping patients and their families to actively participate in the health care process by using evidence-based self-management support strategies;

II *Delivery system design:* creating primary health care teams that deliver and coordinate proactive, preventive and coherent care and support, monitor both the process and quality of care, and guarantee follow-up for patients;

III *Decision support:* using evidence-based treatment protocols and guidelines by professionals and patients by incorporating them into daily practice;

IV *Clinical information systems*: using an electronic patient information system that allows on-site access to essential patient information by professionals and patients, treatment planning and incorporation of guidelines.

These coherent and interacting elements stimulate productive interactions between an informed and activated patient and a prepared, proactive team of professionals, which ultimately could lead to better chronic illness care and improved outcomes [15].

The effects of the CCM have been assessed in numerous studies; however, evidence of the effectiveness of the complete CCM regarding clinical outcomes is scarce [17-19]. The few studies that have implemented the whole model have mainly focused on a specific chronic disease [20]. These studies have demonstrated, among others, improved clinical outcomes for patients with COPD [21], asthma [22], diabetes [23-25], and cardiovascular disease [26]. However, most studies have implemented only a single element or a combination of CCM elements. Although this has already led to demonstrable improvements in health, functional status, and quality of life [18,19,21,27,28], most of these studies lacked a solid study design. Furthermore, the cost effectiveness of the CCM has not been extensively studied [20,29].

The effectiveness of the complete CCM [30], within a population of elderly people that was not defined in terms of any specific disease, was evaluated in a study on the Guided Care model [31]. Effects on outcomes for elderly people and their caregivers [32], quality of care [33], and costs [34] were studied. Patient activation was improved, while caregiver burden was diminished [32]. Also improved coordination of care and positive effects on home health care use were found [33,34]. However, the version of the CCM evaluated in this study was also limited as it focused on a subgroup of elderly people with complex care needs. No studies were found in which the effectiveness of the CCM for the full population of community-living elderly was investigated.

Promotion of healthy ageing for all elderly people is essential in order to prevent disabilities and long-term care needs and to reduce service use in the long run [35]. Furthermore, since the health status of elderly individuals changes frequently and dramatically, those using care today may not use it next year, and vice versa. Consequently, an effective community-based integrated elderly care model should include all elderly people in that community, including the robust elderly [36,37].

To provide a suitable level of care and support for all elderly people in a population, the CCM can be combined with the Kaiser Permanente (KP) Triangle, a population management model for service delivery [19]. The KP Triangle distinguishes three levels with corresponding intervention strategies, based on the distribution of risk for health care needs across a population. At the first level, self-management support is offered to patients at a relatively low risk for health care needs. Patients at the second level have increased levels of risk for complex care needs and receive disease management or care management. The third level consists of high complexity patients, who receive intensive case management. Preventive care is provided at all three levels [17,38].

The aim of the present study is to evaluate the effectiveness of “Embrace” ("SamenOud" in Dutch), a novel population-based integrated elderly care model. Embrace combines the four interacting CCM key elements within the context of the community and health care system with stratification of community-living elderly people in terms of KP-based risk levels. Health outcomes for elderly people and their caregivers, as well as effects on quality of care, service use, and costs will be examined. The present paper will describe the design of the study.

## Methods/Design

### Design and setting

The design of this study is a stratified randomized controlled trial (RCT) with balanced allocation of elderly participants to either the control group or the intervention group. The study will be performed in three municipalities in the province of Groningen, the Netherlands. Municipalities differ in degree of urbanization: rural, urbanized rural, and an industrial municipality. The intervention at the individual level will continue for twelve months and the study will be performed between January 2012 and March 2013. The Medical Ethical Committee of the University Medical Center Groningen has assessed the study proposal and concluded that approval was not required (Reference METc2011.108).

### Study population

Recruitment of the participants will be performed in two steps. First, all general practitioners (GPs) working in the three municipalities will be informed about the study and their consent to participate in the study will be requested. Second, patients from the participating GPs, aged 75 years and older and living at home or in a home for the elderly, are eligible for inclusion in the study and will be invited to participate. Exclusion criteria at baseline are long-term stay in a nursing home, receiving an alternative type of integrated care, or participating in another research study.

Eligible patients will receive a letter from their GP with general information about the intervention and the study performed by the university. One week later, patients will receive a written informed consent form accompanied by questionnaires for baseline measurement (T0). Patients are free to ask for support in filling out the questionnaire, either from family, friends, or from a volunteer available via the project helpdesk. Two and a half weeks later, a reminder will be sent to those patients who have not returned the questionnaire. A further one and a half weeks later, follow-up by telephone will start with respect to the persistent non-respondents. Finally, after inclusion and randomization, participants will be informed by letter about assignment to the intervention or control groups.

Informal caregivers of participants in the intervention group are also eligible for participation in the study. An informal caregiver is defined as a person who is structurally providing voluntary and unpaid care to someone in his/her family, household, or social network with physical, mental, or psychiatric disabilities. During the intervention, caregivers in the intervention group will be invited to participate in the study only after the elderly participant has agreed to their involvement in the study. Caregivers who are willing to participate will receive information about the intervention and study, an informed consent form, and a questionnaire.

### Stratification

Participants will be stratified into three strata, according to the KP Triangle. These strata take into account 1) the complexity of care needs measured with the INTERMED Elderly Self-Assessment (IM-E-SA) [39] and 2) the level of frailty measured with the Groningen Frailty Indicator (GFI) [40], both part of a triage instrument. The strata are: (A) participants without complex care needs and with a relatively low frailty level; (B) frail participants at risk of complex care needs; and (C) participants with complex care needs.

### Intervention: Embrace

Embrace reflects the four key CCM elements: self-management support, delivery system design, decision support, and clinical information systems. Each of these elements will be operationalized to match the population of community-living elderly.

*Self-management support* focuses on the elderly person’s central role in his or her health management. Embrace stresses the elderly person’s perspective on care and support needs. Therefore, effective self-management support strategies will be systematically applied, including shared decision making, motivational interviewing, goal attainment, and action planning. Community meetings for participating elderly individuals will be organized in which the need for prevention and endorsing a healthy lifestyle as well as maintaining self-management abilities will be emphasized.

The *delivery system design* includes Elderly Care Teams (ECTs). These multidisciplinary teams are led by the GP and further consist of an elderly care physician, a district nurse, and a social worker. The focus of the ECTs is on realizing patient centered, proactive, preventive, and coherent care and support taking into account all aspects of functioning and disability, along with environmental aspects. The district nurse or social worker, in the role of case manager, will navigate the elderly person through the complex processes of organizing appropriate care and support in the most efficient, effective, and acceptable way [41]. The GP and elderly care physician will manage the medical care for elderly people with multimorbidity. Monthly ECT meetings will be scheduled, in which (health) problems and treatment options of elderly people and caregivers will be discussed and evaluated. Particularly, attention will be paid to the elderly persons` multimorbidity, polypharmacy, self-management ability, prevention, lifestyle, and future expectations.

*Decision support* will be addressed through multiple decision support tools. A triage instrument is used for stratification (as mentioned above), as well as to provide a suitable level of care and support intensity as offered by the ECT. The second decision-support instrument is a structured history questionnaire based on the results of a Delphi study with a panel of 41 experts (as yet unpublished). This study resulted in a set of 30 categories selected from the International Classification of Functioning, Disability and Health (ICF) [42], regarded as the most common health-related problems of community-living people aged 75 years and older (without dementia). Each topic needs to be assessed by the individual elderly person in the *frail* and *complex care needs profiles.* Assistance in filling out this form will be provided by the case manager during the first home visit. Links to the ICF Browser (for additional categories in the history questionnaire) and to the official guidelines of the Dutch College of GPs (to support evidence-based guidelines) are embedded in the clinical information system.

The *clinical information systems* will be represented by the Electronic Elderly Record System (EERS), a web-based application built for both clinical and research purposes. This computer program is based on work by colleagues who developed a systematic approach to identify patients with complex care needs by scoring the patient and to subsequently systematically document the information [43]. In clinical practice, the EERS will facilitate the ECT members in providing effective care and support. Therefore, the EERS will include personal health records that contain individual triage data, the history questionnaire, an individual care and support plan with information about goal setting, actions performed, and evaluations. For team management purposes, the EERS contains a panel overview and a team agenda. For research purposes, the EERS will be used to evaluate Goal Attainment Scaling (GAS) in the care and support plans, as well as the time expenditures of the ECT members.

### Training

Before the start of the intervention, ECT members will follow an intensive training program that focuses on working according to Embrace, for example, pro-active teamwork, prevention, and working with the EERS. Social workers and district nurses will be trained to fulfill their role as case managers. Furthermore, the case managers will be trained to perform individual and group self-management interventions. GPs will be trained as how to manage their teams in the most effective manner, and to provide care and support that targets specific problems such as multimorbidity, polypharmacy, and dementia. In addition, the project leaders will offer training and support on the job during the monthly ECT meetings, with the focus on the above-mentioned principles and elements. Regular meetings will be organized to exchange ideas and knowledge between ECT professionals and project leaders.

### Embrace profiles: care and support intensity levels

**Figure 1:**
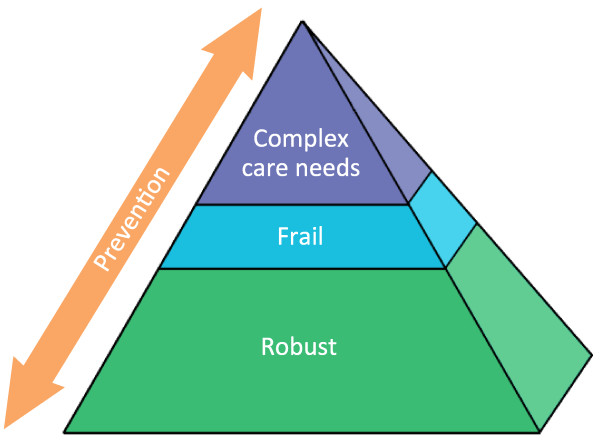
The Embrace Triangle, a population management model for service delivery based on the KP Triangle.

Each stratum corresponds to a so-called Embrace profile: stratum A with the *robust profile*, stratum B with the *frail profile*, and stratum C with the *complex care needs profile* (see Figure 1). Within each of the Embrace profiles, all elements of the CCM will be applied. The profiles differ in care and support intensity levels, as operationalized in the number of contacts with the ECT, the differences in focus of care and support, and the individual or group approach (see Table 1). One of the challenges in Embrace is early detection of changes in health situations and prevention of escalations. Accordingly, proactive adjustments in the care and support intensity level will be made to meet the needs of the individual person, for example, by transferring him or her to another profile, with a corresponding change in the care and support intensity level.

**Table 1:** Characteristics of the Embrace profiles

	**Robust profile**	**Frail profile**	**Complex care needs profile**
**Stratum**	A	B	C
**Level of care and support**	Low-intensity	High-intensity	High-intensity
**Care and support coordination**	ECT	ECT, case manager	ECT, case manager
**Contacts *(no.)***	On patients’ initiative or initiated by ECT	Structured: ±1/month	Structured: ±2/month
**Duration of individual care and support**	Not applicable	6-12 months	6-12 months
**Approach**	Group	Individual	Individual
**Focus**	Self-management	Psychosocial	Health care

The aim for participants in the *robust profile* is to remain healthy and to enhance their self-management capabilities. Attention will be paid to participation in society, for example, by encouraging doing volunteer work and enrolling in courses, combined with education on how to preserve one’s health. Embrace community meetings will be organized to introduce participants to local health care and welfare organizations, courses, activities, and facilities. Participants will be informed about contacting the ECT in case of significant changes in physical or mental health, or in living situations. When a higher level of care and support is needed, the ECT will be consulted and the participant will be transferred to another profile.

Participants in the two other profiles, the *frail* and *complex care needs profiles,* will be visited regularly by their personal case manager. The social worker will offer case management to the frail participants, while the district nurse will offer case management to the participants with complex care needs.

The Embrace process steps for both case managers are as follows: during the first home visit, the case manager will administer the history questionnaire to identify (potential) problems concerning physical function, performance of activities, social participation, and living environment. The severity of the problems identified will be estimated using severity scores ranging from 0 (no problem) to 10 (complete problem). Next, the case manager will formulate a care and support plan in consultation with the participant. This plan is based on the health problems as identified in the history taking, and which are relevant to the participant. Subsequently, for each health problem, goal scores, again ranging from 0 (no problem) to 10 (complete problem), will be estimated and suitable actions selected. The care and support plan will be put into practice after consultation with the ECT, additional examination by the GP or elderly care physician in case of multimorbidity, and after final approval by the participant. During subsequent visits, the case manager will monitor the status of the participant and the implementation of the care and support plan. Changes in medical, social, or living situation of the participant will be monitored and registered. The case manager will keep in close contact with the professionals and volunteers being employed.

The care and support plan will be evaluated regularly and updated and adapted when necessary. Once every four weeks the ECT will discuss the progress in reaching the care and support plan’s goals and the effectiveness of the interventions. Twelve months after the first home visit, all health problems will be evaluated regardless the timeframe, using the goal scores ranging from 0 to 10. The overall health situation will be assessed again using the history questionnaire.

### Care as usual

Participants allocated to the control group will receive care as usual as provided by the GPs and the local health and community organizations. GPs play a central role in the Dutch health care system for those who live in the community and they contribute to an efficient use of resources in their role as gatekeeper by referring patients to specialized medical care. The number of GP visits increases with age from six visits per year at age 45–64 to fifteen visits per year for people aged 75 years and older [44].

### Data collection

Data will be collected with 1) self-report questionnaires for elderly people and self-report questionnaires for professionals before the intervention (T0) and after twelve months (T1), 2) self-report questionnaires for caregivers during the intervention and at T1, 3) from the EERS, and 4) from the financial records of the health insurance company and municipalities involved after finishing the intervention period [45].

### Outcome measurements

The aim of this study is to investigate whether Embrace improves patient outcomes and quality of care in a cost-effective way for all community-living elderly people. Measurement instruments for patient outcomes, quality of care, service use, and costs are summarized in Table 2. Patient outcome measurements will differ per stratum as problems vary; primary and secondary outcomes are chosen according to how sensitive they are to change.

**Table 2:** Primary and secondary outcome measurement instruments for elderly participants per stratum and for caregivers

	**Elderly people**						**Caregivers**		**Professionals**
	**Stratum A**		**Stratum B**		**Stratum C**				
**Measurement instrument**	**Primary**	**Secondary**	**Primary**	**Secondary**	**Primary**	**Secondary**	**Primary**	**Secondary**	
IM-E-SA		P / S		P / S	P	S			
GFI		P / S	P	S	P	S			
GWI		P		P		P			
SMAS-30	P		P			P			
EQ-5D	P / S		P / S		P / S				
Modified Katz ADL		P / S		P / S		P / S			
Quality of life		P		P		P		P	
CSI							P		
MDS-e	S		S		S				
PACIC	Q		Q		Q				
PIH scale		Q		Q		Q			
ACIC									Q^1^
GAS		Q^1^		Q^1^		Q^1^			

P Patient outcome.

Q Quality of care outcome.

S Service use and costs outcome.

^1^ Measured in the intervention group.

### Primary outcome measurements

The primary *patient outcomes* are complexity of care needs, frailty, health status, and self-management ability. The primary caregiver outcome is caregiver burden.

*Complexity of care needs* will be measured using the IM-E-SA. The IM-E-SA measures case complexity with 22 items, divided over four domains: biological, psychological, and social needs, and health care. These domains are approached from three different time perspectives: history, current state, and prognosis [39]. The total score may range from 0 to 60, with a higher score reflecting a higher level of complexity [46].

*Frailty* will be measured using the GFI self-report version. The GFI assesses frailty in the physical, social, cognitive, and psychological domain with fifteen items. The total score may range from 0 to 15, with a higher score indicating a higher level of frailty [40,47].

*Health status* will be measured using the EuroQol-5D (EQ-5D). The EQ-5D is a short self-report questionnaire which measures five dimensions of health by five items on a 3-point scale, ranging from no problems to severe problems: mobility, self-care, usual activities, pain/discomfort, and anxiety/depression. It also includes a Visual Analogue Scale (VAS) to rate the individual’s current health-related quality of life state on a scale ranging from 0 (worst imaginable health state) to 100 (best imaginable health state) [48].

*Self-management ability* will be assessed using the Self-Management Ability Scale (SMAS-30). It contains 30 items which are scored on 5- and 6-point Likert scales. It has six subscales: taking initiatives, self-efficacy beliefs, investment behavior, positive frame of mind, multi-functionality of resources, and variety in resources. The total SMAS score is the average of the six subscale scores, with a higher score indicating better self-management abilities (0–100) [49].

*Caregiver burden* will be evaluated using the Caregiver Strain Index (CSI) which measures both objective and subjective areas of strain: employment, financial, physical, and social areas, and time. It contains thirteen items which are scored on a dichotomous rating scale, with four to six positive responses indicating increased burden and seven or more positive responses indicating a high level of strain [50].

The primary outcomes for *quality of care* are the perceived chronic illness care from the perspective of the participants and their self-management knowledge and behavior.

*Perceived chronic illness care* will be evaluated using the Patient Assessment of Chronic Illness Care (PACIC) [51]. It reflects on all CCM elements and evaluates – from the elderly participants’ perspective – the degree to which integrated care is realized. Originally, the PACIC was developed for people with a specific chronic disease. Since the PACIC will be applied to a population of elderly persons, the disease-related items in the Dutch version of the PACIC [52] will be rephrased into questions more suitable for an ageing population.

*Self-management knowledge and behavior* will be measured with the Partners in Health scale (PIH) [53]. It assesses self-management knowledge and behavior regarding one’s chronic condition. For this study, the English PIH version will be translated into Dutch using forward and backward translation [54] and modified for the elderly population by rephrasing items.

The primary outcome regarding the *cost analysis* is the cost utility expressed in Quality Adjusted Life Years (QALYs) that will be calculated using the health status (EQ-5D) [48] and service use of participants.

*Service use* will be assessed using the Minimal Data Set-economic evaluation (MDS-e) patient self-report questionnaires. This is a set of questions evaluating a wide spectrum of services used during the previous twelve months. The questions focus on hospital stay and ER admissions, nursing-home stay, GP visits, home health care, assist devices acquired, and home adjustments. Standardized cost prices will be used to calculate the costs of services used. If possible, data will be used from databases of health insurers and municipalities.

### Secondary outcome measurements

Secondary *patient outcomes* are well-being, activities of daily living (ADL), and quality of life for both elderly participants and caregivers.

*Well-being* will be measured using the Groningen Well-being Indicator (GWI). This questionnaire measures eight domains of well-being regarding daily experiences: enjoying eating and drinking, sleeping and resting well, having good relationships and contacts, being active, managing yourself, being yourself, feeling healthy in body and mind, and living pleasantly. Respondents have to indicate whether the domains are important to them and, if so, whether they are satisfied with them (as yet unpublished).

*Activities of daily living (ADL)* will be measured using the modified Katz ADL index. This index consists of fifteen items and measures eight physical and seven instrumental ADL. It measures ADL performance by counting the number of disabilities, with a higher score indicating worse functional status (ranging from 0–15) [55].

*Quality of life* of both elderly participants and caregivers will be assessed using two items which are formulated according to the RAND-36 formulation [56]. The first item measures the self-rated quality of life while the second item compares the current self-rated quality of life with quality of life a year earlier.

Secondary outcomes for *quality of care* are the impact of interventions as offered to elderly participants and the perceived chronic illness care from the perspective of the professionals.

*Impact of interventions* will be examined within the intervention group using GAS [57]. GAS is an individualized measure for evaluating the impact of interventions as recorded in the care and support plans of the frail participants and participants with complex care needs.

*Perceived chronic illness care* will be examined within the intervention group using the Assessment of Chronic Illness Care (ACIC) [58]. The ACIC is a professional self-report questionnaire that reflects on all elements of the CCM and evaluates – from the professionals’ perspective – the degree to which integrated care is realized. For this study, the English ACIC version was translated into Dutch using forward and backward translation [54] and modified for the targeted population by rephrasing items.

Secondary outcomes regarding the *cost analysis* will be cost effectiveness, calculated using self-report questionnaires that assess complexity of care needs, frailty, and functional status, in combination with the MDS-e, and – if possible – in combination with data collected from databases of health insurers and municipalities.

### Randomization

**Figure 2:**
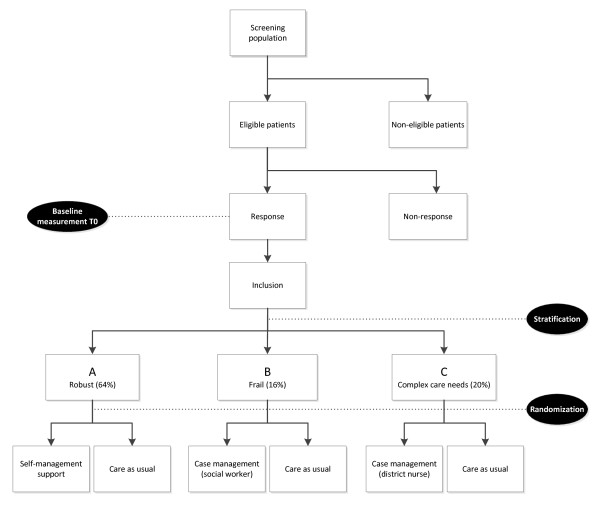
The Embrace study flowchart.

Figure 2 shows the procedure for random assignment to either the intervention or the control group. After stratification, a balancing procedure will be employed per stratum in order to achieve equal distributions of those characteristics that potentially affect intervention outcomes [59]. The balancing criteria will be age, sex, complexity of care needs, frailty, living situation, number of chronic conditions, whether or not receiving homecare, and whether or not receiving help with filling out the questionnaires. This balancing procedure will be performed within each GP practice.

### Sample size

Sample size calculations were based on an expected clinically relevant change per stratum concerning the primary outcome ‘health status’, as measured using the EQ-5D-VAS. To detect a difference of 6 points on the VAS, with a standard deviation of 14 points and a power of 80% (α 2-sided = 0.05), a minimum number of 85 elderly persons are required for the smallest stratum. Based on the results of our pilot study, we estimate that stratum B (frail) will be the smallest stratum: one fifth of the participants at the KP self-care support level had elevated levels of frailty (16%). Consequently, stratum C (robust) will be about 64% (80%-16%), and stratum A (complex care needs) is expected to be around 20%. Taking into account a non-response of 30% and a loss-to-follow-up of 30%, a total number of 2178 elderly people need to be invited to participate.

### Statistical analysis

Descriptive statistics will be used to assess differences between intervention and control groups concerning basic characteristics and confounding variables.

Analyses of the effects regarding primary and secondary outcomes will be executed using the “intention-to-treat principle” [60]. The first analyses will focus on the differences between intervention and control groups after intervention, on the level of the sample and on the level of the strata, with respect to the primary and secondary outcomes. T-tests or (M)ANOVA will be used for continuous variables, Chi square tests, the Wilcoxon tests, and difference of proportions tests for discrete variables. Analysis for change in caregiver burden will focus on the differences between baseline and follow-up measurements by means of Wilcoxon tests, taking into account the duration of support in months. Clinical relevance of the effects will be estimated by Cohen’s effect sizes for statistically significant group differences (p < 0.05).

The incremental cost-effectiveness ratio will be calculated as total costs per QALY [61], and total costs per patient outcome measurement. Bootstrapping will be used to determine the confidence interval. Discounting will initially not be applied considering the limited follow-up period.

For calculation of the GAS, the method proposed by Rockwood [62] will be applied. The first step in this method is to relate the separate problem target scores to the achieved end scores. Next, three specific analyses are performed: 1) calculation of each individual problem score, 2) calculation of overall problem score for each patient, and 3) calculation of the sample score. Finally, the standardized GAS score is used to measure the extent to which the goals were achieved.

The psychometric properties of the PACIC [51] and PIH [53] will be analyzed separately, since these measurements were translated and modified for the current study. For the psychometric evaluation, we will employ confirmatory factor analysis and will estimate the reliability by calculating internal consistency. Criterion validity will be estimated with known-groups comparisons.

### Trial status

The first intervention year has ended and follow-up measurements were completed. Data analyses will start soon.

## Discussion

To our knowledge, the Embrace study is the first RCT that studies the effectiveness, including cost-effectiveness, of an integrated care model that is based on all CCM elements and is applied to a community sample of elderly people.

Previous studies have shown that implementing specific elements of the CCM positively influences outcome variables, but few studies have investigated the effect of applying all elements simultaneously, and most studies concerning the CCM have lacked a solid study design. Furthermore, the CCM has been tested in specific disease populations, but we found no studies in which the effectiveness for the elderly population in general was investigated. In this respect, our Embrace study is unique.

To establish unambiguously the effectiveness of Embrace, a design was developed with a large contrast between intervention and care as usual, with a focus on stratification and blinded randomization of participants, selection of valid and reliable measurement instruments that are sensitive for change, control for confounders, and an adequate sample size. We argue that the current design contains all these.

A few limitations should be mentioned as well. First, the follow-up period may be too short to demonstrate (cost-) effectiveness. Embrace represents a major structural adjustment to an existing system of care, and so requires cultural adaptation. Therefore, it may take time for significant effects in service use and costs to become measurable. This so-called “investment effect” implies that in the first year of an intervention, the health care costs in the experimental group usually are higher than in the usual care group, while intervention benefits will become visible in subsequent years [41,63]. In addition, chronic conditions tend to fluctuate in severity [64]. Multiple measurement moments spread over several years would be preferable. Consequentially, we will attempt to prolong the follow-up period in order to measure the true long-term effectiveness of Embrace.

Another limitation is the risk of selection bias. Elderly people with a poor health status may be less likely to decide to participate [65]. This effect could be reinforced by the length of the questionnaire [66], as well as by the relatively low socioeconomic status and limited (health) literacy in the participating municipalities [67]. Interventions to counteract this risk of selection bias have been built into the data-collection procedure.

Finally, although the design is as strong as possible, we have to deal with potential design bias. Participants will be balanced within GP practices, mainly due to implementation reasons: all participating GPs will be trained in working according to Embrace. Therefore, it is conceivable that participants in the control group will also receive more proactive and preventive care and counseling. However, this risk for contamination is considered to be trivial because the intervention contrasts so strongly with the usual care provided by a GP: a regular GP visit tends to be rather brief and normally takes about 10 minutes [68], with only a small amount of time available for assessment of (potential) health problems or topics [69].

In conclusion, we have started a RCT that will assess the effects of Embrace regarding patient outcomes, service use, costs, and quality of care in a population of community-living elderly. We hope to demonstrate that patient centered, comprehensive, proactive, and preventive care and support, as structured by Embrace, will be a solution to the challenges current health care systems are facing.

## Abbreviations

ACIC: Assessment of Chronic Illness Care; (I)ADL: (Instrumental) activities of daily living; CCM: Chronic Care Model; CSI: Caregiver Strain Index; ECT: Elderly Care Team; EERS: Electronic Elderly Record System; EQ-5D: EuroQOL, health related quality of life, five dimensions; GAS: Goal Attainment Scaling; GFI: Groningen Frailty Indicator; GP(s): General practitioner(s); GWI: Groningen Well-being Indicator; ICF: International classification on functioning, disability and health; IM-E-SA: INTERMED Elderly Self-Assessment; KP-triangle: Kaiser Permanente triangle; MDS-E: Minimal data set economic evaluation; NZa: Dutch healthcare authority; PACIC: Patient Assessment of Chronic Illness Care; PHR: Personal health records; PIH: Partners in Health Scale; QALY: Quality Adjusted Life Years; RCT: Randomized controlled trial; SMAS-30: Self-Management Ability Scale-30; VAS: Visual Analogue Scale; ZonMw: Dutch organization for health research and development.

## Competing interests

All authors declare that they have no competing interests related to this manuscript.

## Authors’ contributions

SLWS and RJU are both principal researchers and drafted and revised this manuscript. KW initiated and coordinates the study. All authors contributed to the design of the study. BM helped in formulating the statistical analyses. SAR and BK are supervising the study. All authors have commented on the manuscript and approved the final version.

## Pre-publication history

The pre-publication history for this paper can be accessed here:

http://www.biomedcentral.com/1471-2318/13/62/prepub
